# A Cytoplasmic New Catalytic Subunit of Calcineurin in *Trypanosoma cruzi* and Its Molecular and Functional Characterization

**DOI:** 10.1371/journal.pntd.0002676

**Published:** 2014-01-30

**Authors:** Patricio R. Orrego, Héctor Olivares, Esteban M. Cordero, Albert Bressan, Mauro Cortez, Hernán Sagua, Ivan Neira, Jorge González, José Franco da Silveira, Nobuko Yoshida, Jorge E. Araya

**Affiliations:** 1 Department of Medical Technology, University of Antofagasta, Antofagasta, Chile; 2 Biomedical Department, University of Antofagasta, Antofagasta, Chile; 3 Department of Microbiology, Immunology and Parasitology, Escola Paulista de Medicina, Universidade Federal de São Paulo, São Paulo, São Paulo, Brazil; 4 Department of Parasitology, Institute of Biomedical Sciences, University of São Paulo, São Paulo, São Paulo, Brazil; Federal University of São Paulo, Brazil

## Abstract

Parasitological cure for Chagas disease is considered extremely difficult to achieve because of the lack of effective chemotherapeutic agents against *Trypanosoma cruzi* at different stages of infection. There are currently only two drugs available. These have several limitations and can produce serious side effects. Thus, new chemotherapeutic targets are much sought after. Among *T. cruzi* components involved in key processes such as parasite proliferation and host cell invasion, Ca^2+^-dependent molecules play an important role. Calcineurin (CaN) is one such molecule. In this study, we cloned a new isoform of the gene coding for CL strain catalytic subunit CaNA (TcCaNA2) and characterized it molecularly and functionally. There is one copy of the *TcCaNA2* gene per haploid genome. It is constitutively transcribed in all *T. cruzi* developmental forms and is localized predominantly in the cytosol. In the parasite, TcCaNA2 is associated with CaNB. The recombinant protein TcCaNA2 has phosphatase activity that is enhanced by Mn^2+^/Ni^2+^. The participation of TcCaNA2 in target cell invasion by metacyclic trypomastigotes was also demonstrated. Metacyclic forms with reduced TcCaNA2 expression following treatment with morpholino antisense oligonucleotides targeted to TcCaNA2 invaded HeLa cells at a lower rate than control parasites treated with morpholino sense oligonucleotides. Similarly, the decreased expression of TcCaNA2 following treatment with antisense morpholino oligonucleotides partially affected the replication of epimastigotes, although to a lesser extent than the decrease in expression following treatment with calcineurin inhibitors. Our findings suggest that the calcineurin activities of TcCaNA2/CaNB and TcCaNA/CaNB, which have distinct cellular localizations (the cytoplasm and the nucleus, respectively), may play a critical role at different stages of *T. cruzi* development, the former in host cell invasion and the latter in parasite multiplication.

## Author Summary

Chagas disease is a neglected tropical parasitic infection. An estimated 10 million people are infected worldwide, and more than 25 million people are at risk of acquiring the disease. The therapeutic agents used to treat the disease may not be effective in all cases and also produce considerable side effects. Therefore, it is important to identify the key factors in the life cycle of the parasite that could be targets for new chemotherapeutic strategies. This paper provides evidence that a new cytoplasmic catalytic subunit of *T. cruzi* calcineurin (TcCaNA2) may play a critical role in host cell invasion by metacyclic trypomastigotes. Metacyclic forms with reduced TcCaNA2 expression following treatment with antisense morpholino oligonucleotides had significantly decreased capacity to invade HeLa cells. Epimastigote proliferation was inhibited to some extent by treatment with an antisense morpholino oligonucleotide targeted to TcCaNA2, but to a lesser degree than by calcineurin inhibitors (CsA, FK506 and INCA-6). The structural differences between TcCaNA2 and its human ortholog CaNA were analyzed to determine the potential of this newly identified calcineurin subunit as a chemotherapeutic target.

## Introduction

Chagas disease, whose etiological agent is *Trypanosoma cruzi*, is a neglected tropical parasitic infection. An estimated 10 million people are infected worldwide, predominantly in Latin America, where it is endemic, and more than 25 million people are at risk of acquiring the disease [1]. The two chemical therapeutic agents used to treat the disease (Nifurtimox and Benznidazole) may cause side effects, and parasitological cure is not achieved in all cases [2], [3]. Identification of key factors in the life cycle of the parasite that could be targets for new chemotherapeutic strategies is therefore very important.

In the life cycle of *T. cruzi*, epimastigote forms replicate in the insect vector and then differentiate into metacyclic trypomastigotes, which are infective to the mammalian host. Cell invasion by metacyclic forms is crucial for the establishment of *T. cruzi* infection. Inside host cells, the parasite replicates as amastigotes, which subsequently transform into trypomastigotes. When the host cell ruptures, these are released to the circulation. There is evidence that Ca^2+^-dependent events are implicated in various processes that are critical for the maintenance of the *T. cruzi* life cycle. It has been shown that the Ca^2+^ chelator EGTA decreases epimastigote multiplication and that intracellular Ca^2+^-concentration increases about six-fold during differentiation of epimastigotes into metacyclic trypomastigotes, an event that is blocked by calmodulin inhibitors [4]. Induction of Ca^2+^ signaling in insect-stage and bloodstream trypomastigotes is an important requirement for target cell invasion [5], [6]. Further, it has been suggested that the Ca^2+^ signal induced in metacyclic forms is associated with the activation of a protein tyrosine kinase [7]. Protein kinases and phosphatases, which control the phosphorylation state of tyrosine, serine and threonine residues, play a pivotal role in cell signal regulation and integration in all living organisms, including trypanosomatids [8], [9]. *T. cruzi* protein phosphatase 2A (PP2A), for instance, has been implicated in the transformation of trypomastigotes into amastigotes [10]. In this scenario, a homolog of mammalian calcineurin has emerged as an important factor for *T. cruzi* infection.

In cells of different tissues, the Ca^2+^-dependent phosphatase calcineurin, also known as PP2B or CaN, is involved in a number of different signaling pathways. An evolutionarily conserved protein in all eukaryotes, it appears to be ubiquitously expressed [11], [12], [13]. It is heterodimeric and consists of calcineurin A (CaNA), the catalytic subunit, and calcineurin B (CaNB), the Ca^2+^-binding subunit [12]. In *T. cruzi* clone CL Brener, Moreno *et al.* [14] identified a protein homologous to CaNA, which is predominantly localized in the nucleus and, unlike its mammalian counterpart, has a catalytic domain and a CaNB-binding domain but lacks the binding domain to calmodulin and the autoinhibitory domain (AID). A protein phosphatase with the same characteristics was also detected in *T. cruzi* CL and G strains, and the sequence of its regulatory subunit (TcCaNB) was determined, revealing the presence of three Ca^2+^-binding domains, known as EF-hand motifs [15]. Treatment of CL strain metacyclic or tissue culture trypomastigotes with CaN inhibitors, such as cyclosporin and cypermethrin, or with antisense phosphorothioate oligonucleotides directed to TcCaNB was shown to inhibit parasite entry into host cells [15]. Whether TcCaN plays other biological functions essential for *T. cruzi* development had not been investigated prior to the present study. We addressed this question and found that TcCaN is also involved in parasite multiplication. In addition, we identified a new isoform of TcCaNA, TcCaNA2 (HM854297), which is localized in the cytoplasm and is implicated in a number of important events, including trypomastigote entry into target cells.

## Materials and Methods

### Ethics statement

All animal handling protocols were performed according to the “Guide for the Care and Use of Laboratory Animals” from the National Institutes of Health, USA [16] and approved by the Institutional Ethics Committee at the Faculty of Health Sciences, University of Antofagasta, Chile (CEIC REV/200) under FONDECYT-Chile grant number 1051045.

### Parasite and cell invasion assay

*T. cruzi* CL strain [17], used throughout this study, was maintained cyclically in Balb/c mice and in axenic liver infusion tryptose (LIT) medium containing 5.0 g liver infusion, 5.0 g tryptose, 4.0 g NaCl, 0.4 g KCl, 8.0 g Na_2_HPO_4_, 2.0 g glucose and 10.0 mg hemin per liter and supplemented with 5% fetal bovine serum. Epimastigote forms were grown at 28°C in LIT medium, and Grace's medium was used to obtain cultures enriched in metacyclic trypomastigote forms, which were purified by chromatography using a diethylaminoethyl (DEAE) cellulose column (Sigma Chemical Co), as described by Teixeira *et al.* [18]. Cell invasion assays were performed as described by Ramirez *et al.* [19]. Briefly, 2×10^5^ HeLa cells were cultured in 4-well Lab-Tek Chamber Slides (Nunc, Thermo Scientific). After adhesion and growth at 37°C in a humidified 5% CO_2_ atmosphere, the cells were incubated with 1×10^6^ metacyclic trypomastigotes (MT) previously treated or not with different calcineurin inhibitors. After 3 h incubation, the cells were washed with PBS and fixed with methanol followed by Giemsa staining. The number of intracellular parasites was counted in 100 cells. Assays were conducted in triplicate. The viability of MT was evaluated by Trypan blue exclusion and by parasite migration assay through gastric mucin layer. Briefly, polycarbonate transwell filters (3 mm pores, 6.5 mm diameter, Costar) were coated with 50 µL of a preparation containing 10 mg/ml gastric mucin. *T. cruzi* metacyclic trypomastigotes, in 600 µL PBS were added to the bottom of 24-well plates (1×10^7^ parasites/well) and incubated for 1 h at 37°C. Thereafter, the mucin-coated transwell filters were placed onto parasite-containing wells, and 100 µL PBS were added to the filter chamber. After 1 h of incubation at 37°C, 10 µL were collected from the filter chamber for determination of parasite number and the volume in this chamber was corrected by adding 10 µL PBS [20].

### Parasite proliferation inhibition assay

Epimastigotes maintained in LIT medium were used for proliferation assays. Parasites in growth phase were incubated with different concentrations of calcineurin inhibitor cyclosporin A (CsA). Untreated or treated parasites with ethanol were used as controls. Assays were performed with three different cultures, each containing 5×10^5^ epimastigotes per sample in a volume of 1 mL of LIT medium. Parasite cultures were analyzed daily for one week by taking samples to measure the number of parasites using a Neubauer hemocytometer, and the viability of epimastigotes was determined under light microscopy using Trypan blue exclusion and CFDA-SE assays [21]. Results were expressed as mean ± standard error of three independent experiments. Similarly, epimastigotes were incubated with different concentrations of calcineurin inhibitors [CsA, tacrolimus (FK-506), INCA-6 and kaempferol (Kmp)] at 0, 10, 20 and 40 µM. Crystal violet was used as a positive control because of its trypanocidal effect. Assays were performed in triplicate in 96-well microplates containing 5×10^5^ epimastigotes per well in 200 µL of LIT medium. After 72 h incubation, the number of parasites was counted as described above. Untreated epimastigote cultures were used as a negative control. Results were expressed as a percentage of proliferation (number of cells) in the control group.

### Isolation of nucleic acids, Southern and Northern blot hybridizations and separation of chromosomal bands

Isolation of genomic DNA and total RNA from *T. cruzi* CL strain, Southern and Northern blot hybridizations and separation of chromosomal bands by pulsed-field gel electrophoresis were performed as described previously [10].

### Cloning of *T. cruzi* CaNA2 and CaNB genes

To clone the CaNA2 gene of *T. cruzi* CL strain, sense and antisense primers were designed based on the sequence of the genome of CL Brener clone available in GenBank (http://www.ncbi.nlm.nih.gov) under accession number XM_816360.1. Sense primer 5′-ATG TTG TCT ACA TCA GAT TCT-3′ and antisense primer 5′-TCA TTT GCA TCC CTT ATT TAG-3′ were used. After amplification of the TcCaNA2 gene by RT-PCR with 1–20 ng of *T. cruzi* cDNA synthesized using poly A+ mRNA (using oligo dT) obtained from epimastigotes, PCR products were analyzed by agarose gel electrophoresis. The amplification product was cloned in the vector pCR 2.1-TOPO (TOPO Cloning Vector Kit for sequencing, Invitrogen by Life Technologies) according to the manufacturer's instructions. The CaNB gene was amplified by RT-PCR using a pair of primers: sense 5′-CGG AAT TCA TGG GCG AGG GGG T-3′ and antisense 5′-CGG AAT TCC TAA ATG GAG AGG C-3′, which were based on a cDNA sequence from the CL strain (accession number AY570505). A cloning protocol similar to that described for the TcCaNA2 gene was used. Sequences were analyzed using DNASTAR and GeneDoc software and National Center for Biotechnology Information (NCBI) programs (http://www.ncbi.nlm.nih.gov).

### Expression and purification of recombinant proteins TcCaNA2 and TcCaNB

The coding sequences of TcCaNA2 and TcCaNB genes were subcloned into the expression vector pGEX-1λT (GE Healthcare) in-frame with glutathione S-transferase (GST) gene. After sequencing the construct to check that the open reading frame was in the correct orientation, expression of the recombinant protein was induced in *E. coli* BL21 (DE3) after addition of 1 mM isopropyl thio-β-D-galactoside (IPTG). The recombinant protein was purified by the cleared lysate method using gluthathione-Sepharose 4B (Amersham Biosciences). After washing with PBS pH 7.3, the proteins of interest were eluted with 200 mM Tris-HCl pH 8.0, 40 mM reduced glutathione, 150 mM NaCl, 5 mM DTT and 0.1% Triton X-100. Analysis of the purified protein was carried out by SDS-PAGE on 10% gels stained with Coomassie blue. Additionally, the coding sequence of TcCaNA2 was cloned in-frame into the expression vector pET-SUMO (Champion pET Expression System, Invitrogen by Life Technologies). The recombinant protein was expressed in *E. coli* BL21 (DE3) after induction with IPTG, and the protein (6×His-SUMO-TcCaNA2) was purified from cleared lysates by affinity chromatography on Ni-NTA agarose (Invitrogen by Life Technologies). After elution in the presence of imidazole (SIGMA), the purity of the protein was determined as above.

### Determination of enzymatic activity of TcCaN and TcCaNA2

To measure TcCaN activity, parasites were washed three times with TBS (150 mM NaCl, 20 mM Tris, pH 7.2) and then lysed in lysis buffer (50 mM Tris pH 7.5, 1 mM DTT, 100 µM EDTA, 100 µM EGTA, 0.2% NP-40) and centrifuged at 100,000× *g* for 45 min at 4°C. The high-speed post-lysis supernatants were used to evaluate calcineurin-type phosphatase activity with a Calcineurin Cellular Activity Assay Kit, Colorimetric (Calbiochem, USA), according to the manufacturer's instructions. To determine the phosphatase activity of TcCaNA2, the assay was performed using 1 µg of the recombinant protein in the presence of one of the following metal ions at 1 mM: CaCl_2_, MgCl_2_, MnCl_2_ or NiCl_2_. The recombinant TcCaNA2 was incubated for 15 min at 30°C with 80 mM *p*-nitrophenyl phosphate (*p*-NPP) (Calbiochem, USA) as substrate. The reaction was stopped by adding 950 µL of 1 M NaOH, and enzyme activity was measured by the change in absorbance at 405 nm, as described by Sagoo *et al.* [22]. Similarly, the activity of 1 µg of TcCaNA2 combined with 1 µg of TcCaNB was measured in the presence of 1 mM of MnCl_2_ with or without EGTA.

### Generation and purification of antibodies

Two protocols were developed to generate antibodies. First, BALB/c mice and/or New Zealand white rabbits (after obtaining pre-immune serum) were immunized with GST-TcCaNA2 and GST-TcCaNB recombinant proteins. Each animal received 4 doses of 10 µg antigen plus 0.5 mg Al(OH)_3_ as adjuvant at 7-day intervals. After the last immunization dose, blood was obtained by cardiac puncture. The polyclonal antiserum was divided into aliquots and stored at −20°C in the presence of 0.1% sodium azide as preservative. Monospecific polyclonal antibodies against the TcCaNA2 isoform were raised in New Zealand white rabbits by immunization with the synthetic peptides 246–264 (CGSKSDYYTPSAGPSYGSKP-amide) and 206–224 (Ac-IKLNHIDLIHRFRE PPSRGC-amide) conjugated to KLH (*Keyhole limpet* hemocyanin) or ovalbumin using MBS (m-Maleimidobenzoyl-N-hydroxysuccinimide ester). The monospecific antibodies were purified by affinity chromatography using the peptide 246–264, which is only present in the TcCaNA2 isoform, as ligand.

### Far-Western blot analysis

To evaluate the interaction between TcCaNA2 and TcCaNB, a Far-Western blotting was performed as described by Wu *et al.* [23] using the recombinant proteins TcCaNB (tagged with GST) and TcCaNA2 (tagged with 6×His). BSA and the unrelated fusion protein 6×His-SUMO-CAT were used as negative controls. One microgram each of BSA, CAT and 6×His-SUMO-TcCaNA2 (target protein) were resolved in 10% SDS-PAGE and transferred onto PVDF membranes, which were then incubated with decreasing concentrations of guanidine-HCl (6, 3, 1, 0.1, and 0 M) to denature and renature the target protein. The membrane was then blocked with PBS containing 0.5% Tween 20 and 5% skim milk and incubated with 10 µg of the bait protein GST-TcCaNB. A rabbit polyclonal antibody directed to GST-TcCaNB and an anti-rabbit IgG antibody conjugated to horseradish peroxidase (Sigma-Aldrich) were used to detect TcCaNA2-TcCaNB interaction. The immunocomplexes were revealed using diaminobenzidine (DAB) as substrate.

### Western blot for detection of TcCaNA2 in parasite extracts

Epimastigotes (1×10^7^) were lysed in Laemmli buffer (62.5 mM Tris-HCl pH 6.8, 25% glycerol, 2% SDS, 0.01% bromophenol blue) and exposed to different concentrations of urea (0, 2, 4 and 6 M) overnight at 4°C to stabilize the solutions, which were then separated on 10% SDS-PAGE gels, electrotransferred to a PVDF membrane (Amersham Hybond-P) and visualized by Western blot.

### Antisense oligonucleotides (AS-ONs, morpholino type) directed to TcCaNA2

The antisense oligonucleotide (AS-ON) used was 5′- GAG AAT ATG CTG TAG ACA ACA TTA T-3′, and the AS-ON control was a standard unrelated oligonucleotide: 5′-CCT CTT ACC TCA GTT ACA ATT ATT-3′. The assays were performed in the presence of antisense oligonucleotides directed to TcCaNA2 (third generation morpholino, Gene Tools, LLC), and the effects were evaluated as described above for the assays used to detect inhibition of cell proliferation and invasion. Each AS-ON was added at a concentration of 10 µM with 6 µM of Endo-Porter, according to the Gene Tools “Endo-Porter delivery of morpholino oligos” protocol.

### Determination of the subcellular distribution of TcCaNA2

Epimastigotes in exponential growth phase (CL strain) were centrifuged at 6,000× *g* for 5 min, washed twice in PBS pH 7.2 and then resuspended at 2×10^6^ parasites/mL. Parasites were decanted on poly-L-lysine-treated cover slips and fixed in PBS containing 4% paraformaldehyde. Next they were permeabilized with PBS-0.5% Triton X-100 for 5 min at room temperature and blocked with a solution of 2% glycine, 2% BSA, 5% FCS and 50 mM NH_4_Cl in PBS pH 7.2. The cover slips were then incubated with the primary antibodies, washed three times for 5 min in PBS pH 7.2 and incubated with the secondary antibodies (anti-mouse Alexa Fluor 488 and anti-rabbit Alexa Fluor 568). The samples were visualized with a reflected light microscope (Carl Zeiss, model Axiovert 10), photographed and analyzed using QCapture Pro 6.

### Statistical analysis

Statistical analysis was carried out with GraphPad Prism v. 5.0 (GraphPad Software, Inc., San Diego, CA). Values of p<0.05 were considered significant.

## Results

### Effect of CaN inhibitors on *T. cruzi* proliferation

**Figure 1:**
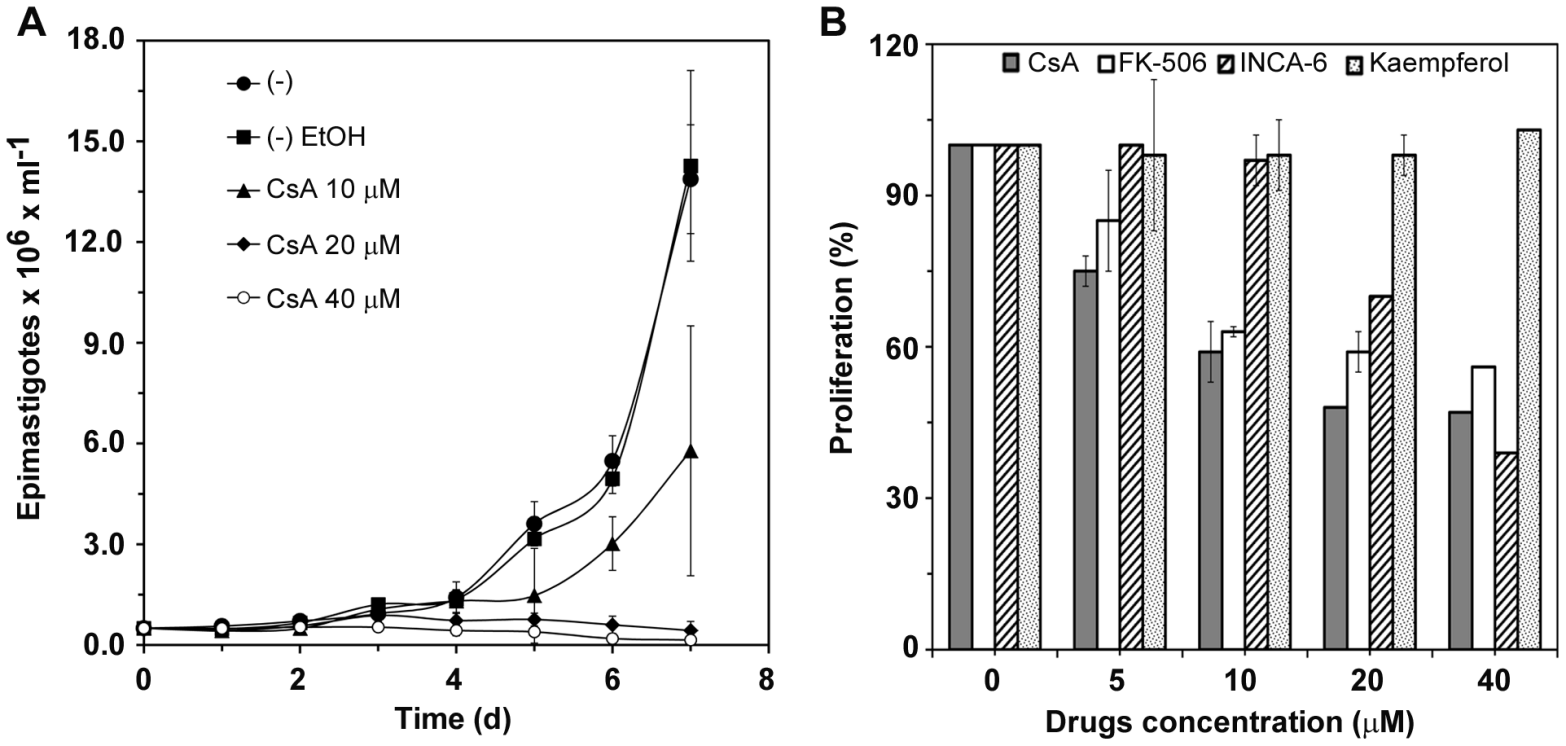
Effect of calcineurin inhibitors on *T. cruzi* proliferation. A) Epimastigotes were incubated with different concentrations of CsA (10, 20 and 40 µM). Untreated parasites or parasites treated with ethanol were used as a control. Assays were performed in three different cultures, each containing 5×10^5^ epimastigotes per sample in 1 mL of LIT medium supplemented with 10% FBS. Cultures were monitored daily for one week to determine parasite density. B) *T. cruzi* epimastigotes were maintained for 72 hours in LIT medium supplemented with 10% FBS in the absence (control) or with different concentrations (5, 10, 20 and 40 µM) of the following calcineurin inhibitors: CsA, FK-506, INCA-6 and kaempferol. Cell proliferation was calculated as a percentage of the number of parasites in the control group. Results are expressed as mean ± standard error of three independent experiments performed in triplicate.

It has previously been shown that the treatment of CL strain metacyclic trypomastigotes or tissue culture-derived trypomastigotes with calcineurin inhibitors CsA or cypermethrin resulted in strong inhibition (62–64%) of parasite entry into HeLa cells [15]. To determine the role of TcCaN in parasite growth, epimastigotes were incubated in the absence or presence of varying concentrations of the calcineurin inhibitor CsA. Starting on the day CsA was added, parasite density was measured daily for one week, and it was observed that parasite growth was inhibited by CsA. At 20 µM and 40 µM, CsA inhibited parasite growth completely whereas at 10 µM inhibition was partial (Figure 1A). Epimastigotes treated with CsA for 24 h remained viable and were indistinguishable from the control untreated parasites (morphology and motility), although their growth was impaired. Loss of viability was observed when epimastigotes were treated with CsA for 48 h or longer (data not shown). Other calcineurin inhibitors, such as FK-506 and INCA-6, were also tested, with similar results (Figure 1B). Among the inhibitors tested, CsA and FK-506 were more effective than INCA-6 to inhibit parasite proliferation. No inhibition was observed after treatment with kaempferol (Figure 1B). These data indicate that TcCaN plays an important role at different stages in *T. cruzi* development.

### Isolation and characterization of the gene encoding a new catalytic subunit of *T. cruzi* CaN (TcCaNA2)

The nucleotide sequence corresponding to a gene coding for the catalytic subunit of the CL strain CaN, which is distinct from that previously reported [15] and was therefore called TcCaNA2, was obtained by RT-PCR amplification using primers based on a genomic sequence of clone CL Brener and deposited in GenBank under accession number HM854297. In silico analysis using BLASTp showed 44% identity between TcCaNA2 and CL strain TcCaNA (accession number EU195113), described by Araya *et al.* [15], and between TcCaNA2 and the catalytic subunit A (CnA-like: accession number AJ878872), described by Moreno *et al.* [14] for CL Brener clone.

**Figure 2:**
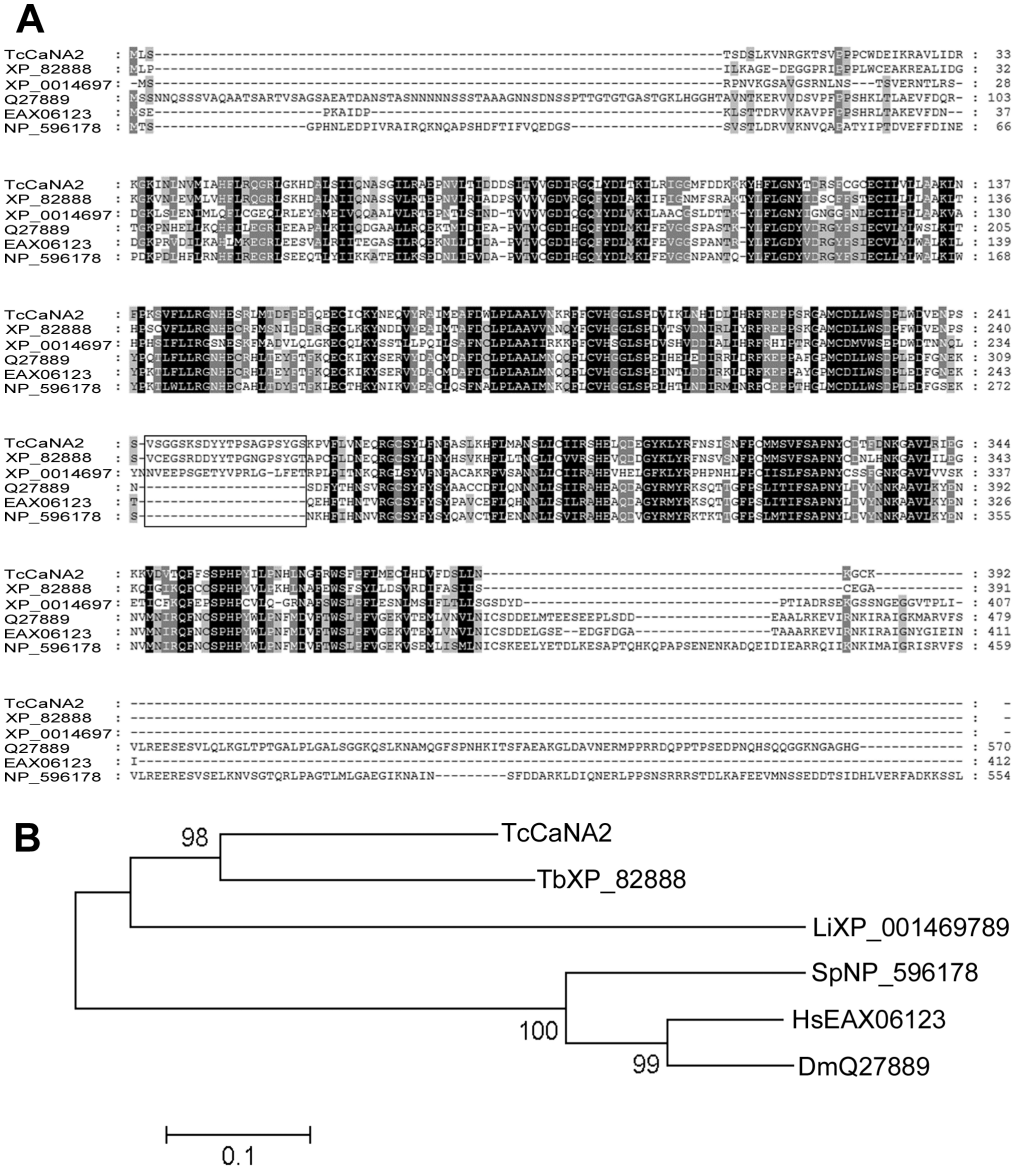
Comparison of TcCaNA2 amino acid sequence with catalytic subunits from different species. A) Alignment of *T. cruzi* CL strain TcCaNA2 (HM854297) with CaNA from *Trypanosoma brucei* TREU927 (TbXP_822888.1), *Leishmania infantum* (LiXP_001469789.1), *Schizosaccharomyces pombe* 972 h (SpNP_596178.1), *Drosophila melanogaster* (DmQ27889.2) and the alpha isoform of *Homo sapiens* (HsEAX06123.1). Genbank accession numbers are in parentheses. Conserved residues are in black (100% conservation), dark gray (**>**75% conservation) and light gray (**>**50% conservation); no shading denotes residues with **<**50% conservation. The peptide sequence 243-VSGGSGSDYYTPSAGPSYGS-262 is marked with a line within the box. B) Phylogenetic tree inferred from the amino acid alignments of TcCaNA2-CL with other CaNAs. Phylogenetic analysis was performed with MEGA4 software [71]. Evolutionary history was analyzed using the Neighbor-Joining method [72] with 1000 bootstrap replicates [73]. Evolutionary distances were calculated using the Poisson correction method [74] and are in units of number of amino acid substitutions.

The TcCaNA2 nucleotide sequence showed an open reading frame (ORF) of 1179 bp, which codes for a polypeptide of 392 amino acids with an estimated molecular mass of 44.5 kDa. The deduced amino acid sequence of TcCaNA2 possesses the domain that interacts with CaNB, but the calmodulin-binding domain and AID are absent (Figure 2A). The conserved GDXHG, GDXVDRG and GNHE (phosphoesterase) motifs, which are characteristic of serine-threonine protein phosphatases, are present (Figure 2A). A strong homology between TcCaNA2 and CaNA from other species was found: 44% identity with *Homo sapiens* and 45% with *Drosophila melanogaster* and *Neurospora crassa*. Among trypanosomatids, TcCaNA2 showed 46% and 67% identity with CaNA from *Leishmania major* and *Trypanosoma brucei*, respectively.

The neighbor-joining cladogram revealed two major clusters, one of which contains CaNA from three trypanosomatids: *T. brucei* TREU927 (XP_822888.1), *Leishmania infantum* (XP_001469789.1) and *T. cruzi* (TcCaNA2-CL, HM854297). This shows the ancestral relation of these organisms and the closer relationship between the two *Trypanosoma* species (Figure 2B); the CaNA2 gene of *T. cruzi* is the most conserved among the species analyzed. The second cluster included *Schizosaccharomyces pombe* 972 h- (NP_596178.1), *D. melanogaster* (Q27889.2) and the alpha isoform of *H. sapiens* (EAX06123.1), showing an evolutionary variation among species (Figure 2B).

### Genomic organization and transcription of TcCaNA2

A BLASTn search [http://www.genedb.org; Gish, W. (1996–2006); http://blast.wustl.edu] retrieved the 21178 bp contig 8328, which contains one copy of the TcCaNA2 gene; a query in the kinetoplastid genome database (2012 The EuPathDB Project Team; http://tritrypdb.org/tritrypdb/) showed that the TcCaNA2 gene is located on chromosome TcChr37P, which belongs to the non-Esmeraldo haplotype of CL Brener clone. However, no sequence similarity was found within chromosome TcChr37S from Esmeraldo haplotype (data not shown).

**Figure 3:**
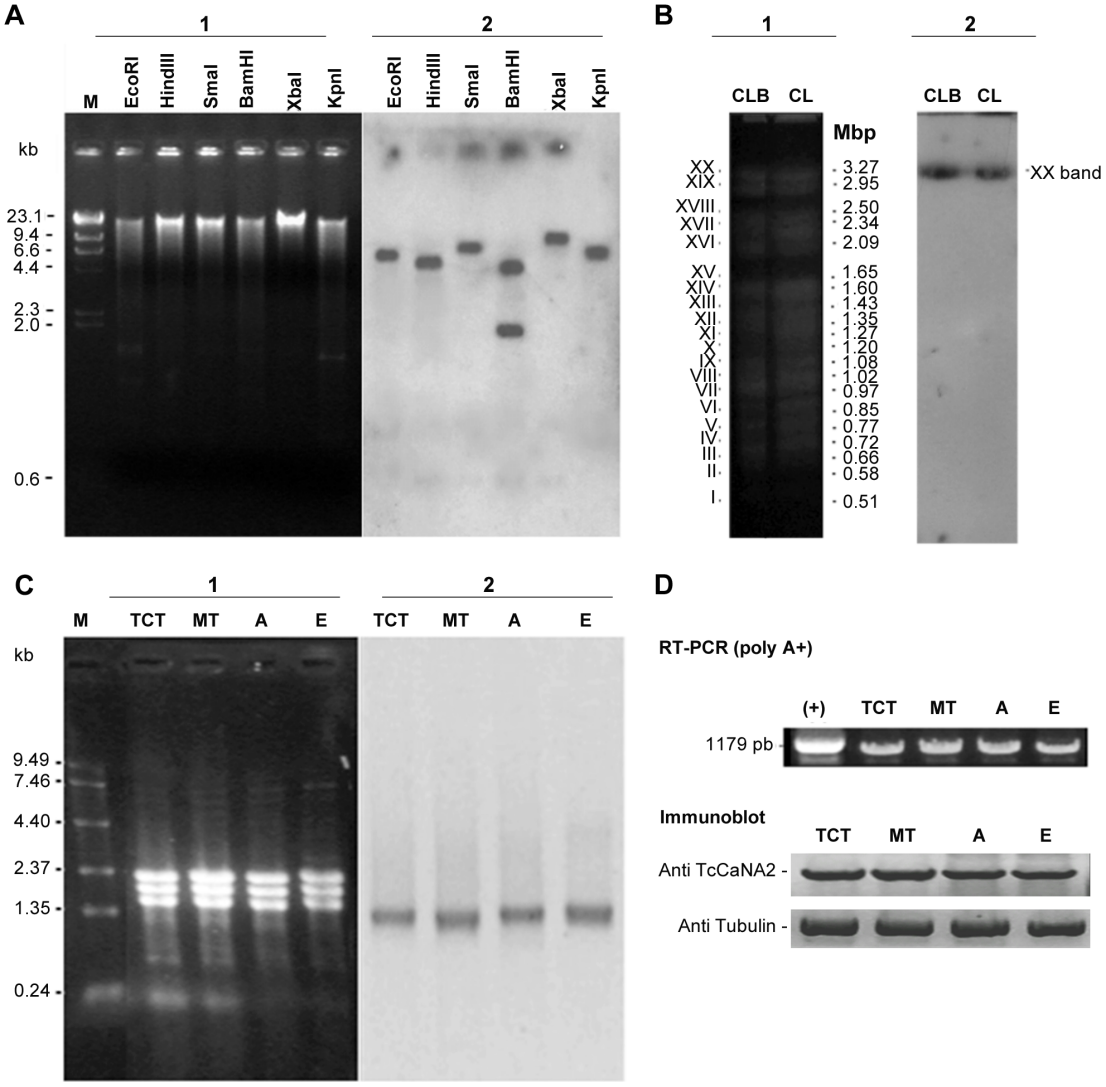
Genomic organization and transcription of *T. cruzi* CaNA2 gene. A) Southern blot hybridization. CL strain genomic DNA was digested with the indicated restriction enzymes, electrophoresed in agarose gel (1), transferred to Hybond-N membrane and hybridized to the ^32^P-labeled TcCaNA2 gene probe (2). Size markers are indicated in kilobases (kb). B) Chromoblot hybridization. Chromosomal bands of epimastigotes from clone CL-Brener (CLB) and CL strain (CL) were separated by PFGE, stained with ethidium bromide (1), transferred to Hybond-N membrane and hybridized with the same probe described above (2). Size markers are indicated in megabase pair (Mbp). C) Northern blot hybridization. Total RNA (12 µg) extracted from different developmental forms of *T. cruzi* CL strain (TCT: tissue culture trypomastigotes, MT: metacyclic trypomastigotes, A: amastigotes, E: epimastigotes) was subjected to formaldehyde-agarose gel electrophoresis, stained with ethidium bromide (1), transferred to Hybond-N membrane and hybridized with the same probe described above (2). M, molecular size markers, are indicated in kilobases (kb). D) Amplification of TcCaNA2 gene by RT-PCR (upper) and immunoblotting of TcCaNA2 protein expression (lower) in different developmental forms of *T. cruzi*. M, molecular size markers (kb). (+): positive control, corresponds to plasmid pCR 2.1-TOPO with TcCaNA2.

Southern blot analysis of CL strain genomic DNA showed a simple pattern of hybridization with a probe derived from TcCaNA2 gene, with two internal *Bam*HI restriction sites (Figure 3A). The same probe revealed only one chromosomal band (chromosomal band XX ∼3.27 Mb) in clone CL-Brener and CL strain chromosomes resolved in pulsed field gel electrophoresis (Figure 3B). This hybridization profile suggests that a single copy of TcCaNA2 gene is present, although the presence of two co-migrating chromosomal bands cannot be ruled out. Northern blot hybridizations showed a transcript of approximately 1.5 kb in all developmental stages of the parasite (Figure 3C), indicating that the transcription of TcCaNA2 is constitutive. Densitometric analysis on the same autoradiogram hybridized with a tubulin-derived probe confirmed equal loading of the samples (data not shown). Similarly, using a pair of primers flanking the ORF of the TcCaNA2 gene, an RT-PCR on cDNA freshly synthesized from parasite mRNA revealed amplification in all developmental forms (Figure 3D), confirming what was observed in the Northern blot (Figure 3C). Correspondingly, TcCaNA2 protein was also detected in all developmental stages (TCT, MT, A and E), being the protein levels slightly greater in MT and TCT, as demonstrated by immunoblotting (Figure 3D).

### Enzymatic activity of TcCaNA2 and the effect of metal ions

**Figure 4:**
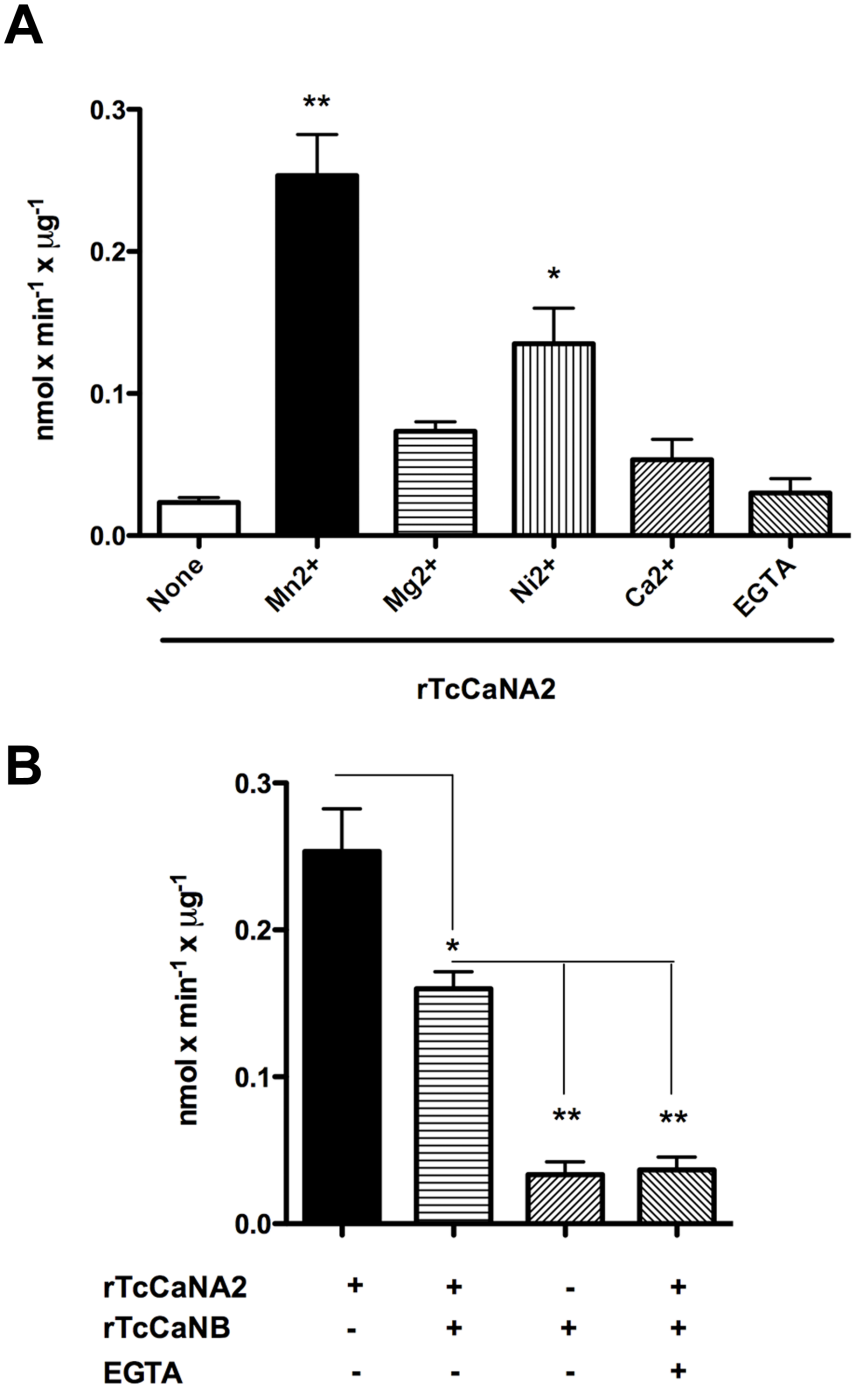
Effect of metal ions on the phosphatase activity of rTcCaNA2 and TcCaNA2/TcCaNB. A) Catalytic activity was evaluated in the presence of the divalent cations at 1 mM, using 80 mM *p*-NPP as substrate and 1 µg rTcCaNA2. All data are presented as means ± standard error of the mean (SEM) of triplicates. Statistical significance was determined by Tukey's Multiple Comparison Test (*p*<0.05). **p = 0.0015 and *p<0.05 in relation to the activity without metal ions. The results are representative of two independent experiments performed in triplicate. B) Catalytic activity of TcCaNA2 was evaluated as in A in the presence of rTcCaNB and/or EGTA. Statistical significance was determined by Tukey's Multiple Comparison Test (*p*<0.05). *p<0.05 in relation to the activity with only rTcCaNA2 and **p<0.05 in relation to activity of rTcCaNA2/rTcCaNB heterodimer. The results are representative of two independent experiments performed in triplicate.

The activity of the purified recombinant TcCaNA2 was determined after its purity and specific recognition by polyclonal monospecific anti-TcCaN2 antibody had been confirmed. Recombinant TcCaNA2 was incubated in the absence or presence of 1 mM CaCl_2_, MgCl_2_, MnCl_2_ or NiCl_2_, and the reaction using *p*-NPP as substrate proceeded for 15 min at 30°C. Absorbance reading at 405 nm revealed higher TcCaNA2 activity in the presence of Mn^2+^ or Ni^2+^, indicating that these metal ions are more effective cofactors than Ca^2+^ or Mg^2+^ (Figure 4A). Our data showing that the recombinant TcCaNA2 is activated by Mn^2+^/Ni^2+^ (Figure 4A) are compatible with the report suggesting that enzyme activation by Mn^2+^/Ni^2+^ is mainly mediated via the catalytic domain, if it is assumed that the non-catalytic domains of subunit A negatively regulate the activity of calcineurin by acting as intra-molecular inhibitors [24]. As TcCaNA2 interacts with TcCaNB *in vivo*, the activity of TcCaNA2 combined with TcCaNB was measured, by adding 1 µg of each protein recombinant. TcCaNA2 activity did not depend on its association with TcCaNB (Figure 4B). Taking into account that the specific activity is expressed as nmol per min per µg protein, the reduced activity of TcCaNA2, when combined with TcCaNB, is only apparent and is due to the presence of 2 µg protein. Activity of TcCaNA2/TcCaNB was significantly inhibited by EGTA (Figure 4B).

### Interaction between recombinant TcCaNA2 and TcCaNB proteins and the effect of urea on dissociation of native TcCaNA2/TcCaNB

**Figure 5:**
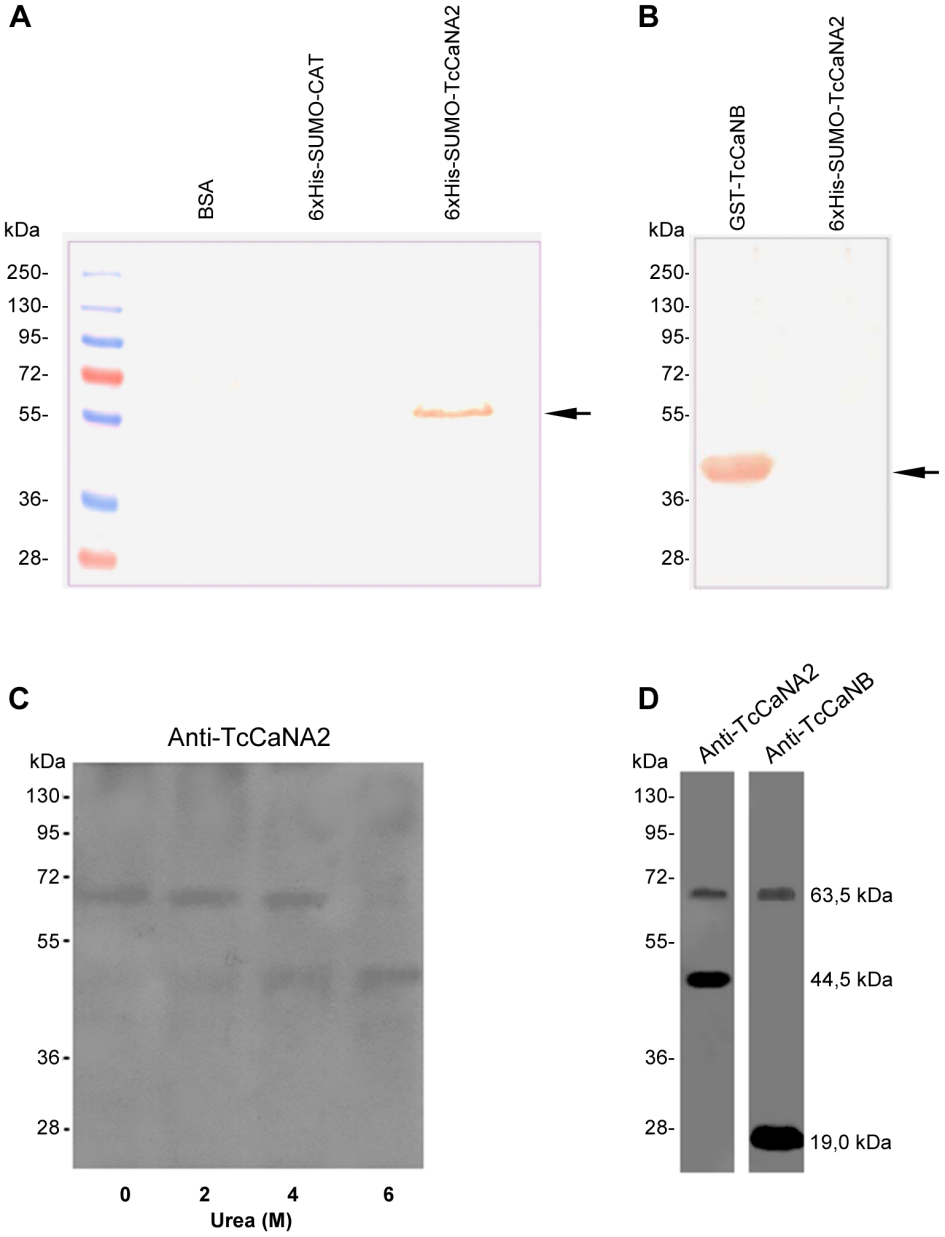
Interaction between TcCaNA2 and TcCaNB and the effect of urea on the dissociation of native TcCaNA2/TcCaNB. A) Far-Western blotting was used to detect interaction between recombinant TcCaNA2 and TcCaNB proteins. BSA (66.5 kDa) and the recombinant proteins 6×His-SUMO-CAT (39.0 kDa) and 6×His-SUMO-TcCaNA2 (55.7 kDa) were subjected to SDS-PAGE and electroblotted onto a PVDF membrane. Proteins were denatured and renatured and subsequently incubated with purified GST-TcCaNB (45.0 kDa) protein. The arrow indicates the position of 6×His-SUMO-TcCaNA2 (55.7 kDa). B) GST-TcCaNB and 6×His-SUMO-TcCaNA2 proteins were subjected to SDS-PAGE and electroblotted onto a PVDF membrane, which was then incubated with the anti-GST-TcCaNB antibodies to ascertain specific recognition of TcCaNB. The arrow indicates the position of GST-TcCaNB. C) Total protein extracts from epimastigotes (1×10^7^) were treated at 4°C with urea at the indicated concentrations, subjected to SDS-PAGE and electroblotted onto PVDF membrane, which was reacted with anti-TcCaNA2 monospecific polyclonal antibody. D) Immunoblotting of total protein extracts of *T. cruzi* epimastigotes treated with 4 M urea using monospecific polyclonal antisera directed toward TcCaNA2 (rabbit) and TcCaNB (mouse).

To confirm the molecular interaction between TcCaNA2 and TcCaNB, we performed a Far-Western blot assay as described by Wu *et al.* [23]. The recombinant proteins GST-TcCaNB and 6×His-SUMO-TcCaNA2 were used as bait and target, respectively. As negative controls, BSA and the unrelated fusion protein 6×His-SUMO-CAT were used. Binding of GST-TcCaNB to 6×His-SUMO-TcCaNA2 was shown using the mouse polyclonal anti-TcCaNB antibody and anti-IgG tagged with horseradish peroxidase. As shown in Figure 5A, TcCaNB interacts with TcCaNA. We ascertained that anti-TcCaNB antibody specifically reacts with TcCaNB and does not recognize 6×His-SUMO-TcCaNA2 (Figure 5B). In addition, to show that native TcCaNA2 and TcCaNB are associated in the parasites, we performed an assay to detect the dissociation of the two subunits. Total extracts from epimastigotes (1×10^7^) were exposed to different concentrations of urea at 4°C, subjected to SDS-PAGE and electroblotted onto PVDF, and TcCaNA2 was revealed using anti-TcCaNA2 antibody and peroxidase-conjugated IgG, followed by ECL. In the absence of urea, a band of approximately 63.5 kDa was detected (Figure 5C) that is compatible with the size of TcCaNA2/TcCaNB assuming that the molecular mass of TcCaNA2 is 44.5 kDa and that of TcCaNB 19 kDa, as determined by Western blot using antibodies specific to these subunits (Figure 5D). After treatment of parasite extracts with urea, there was a change in this profile. A component of about 45 kDa appeared as a weak band in the sample treated with 2 M of urea, had its intensity increased with 4 M of urea and was the sole band detected by anti-TcCaNA2 antibody (Figure 5C). We inferred from this observation that TcCaNA2 and TcCaNB are closely associated in *T. cruzi*.

### Cytosolic localization of TcCaNA2 in *T. cruzi* and CaN activity in high-speed cytosolic extracts

**Figure 6:**
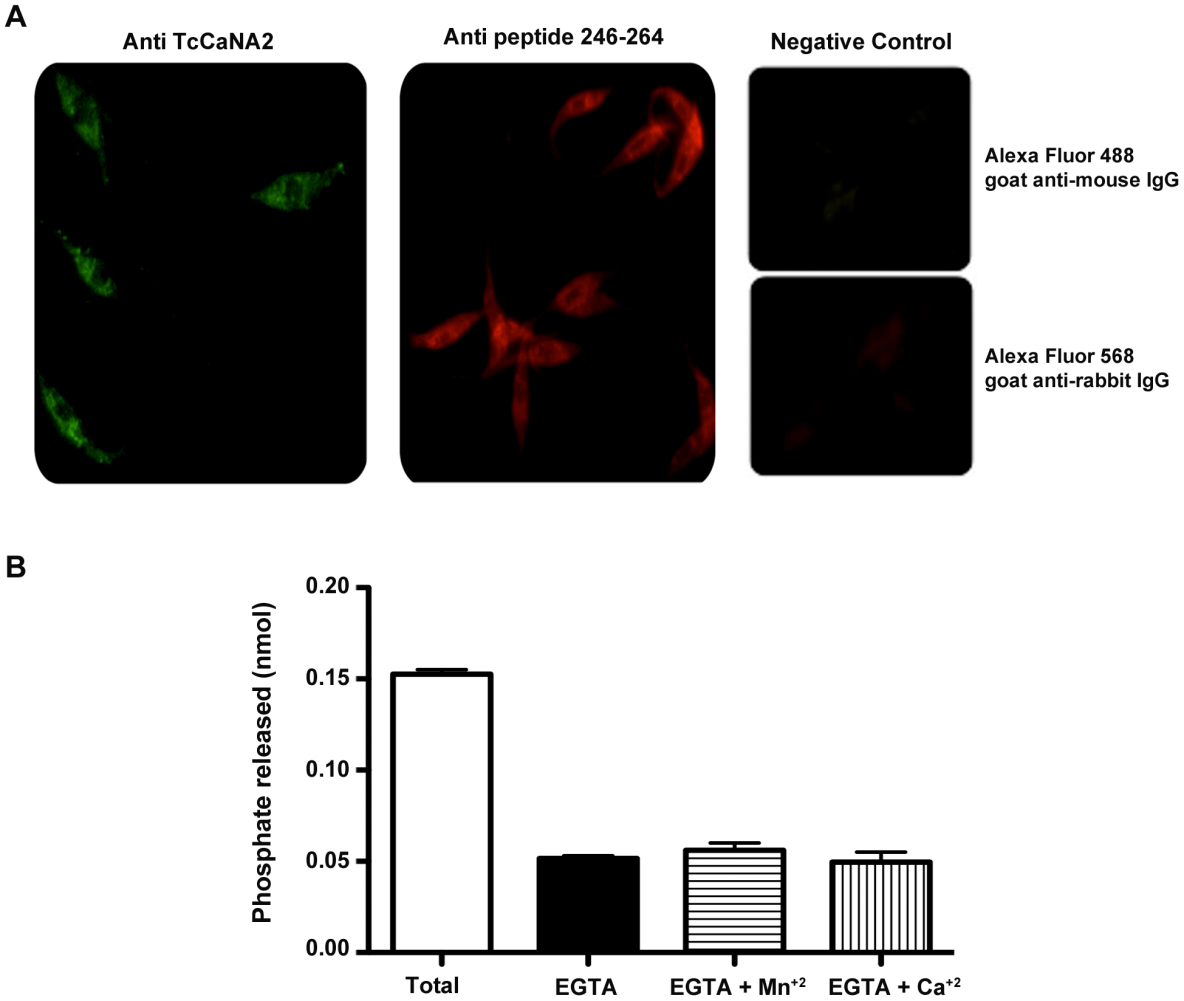
Cellular localization of *T. cruzi* TcCaNA2. A) Epimastigotes were fixed and incubated with mouse polyclonal antibodies raised against recombinant TcCaNA2 or with rabbit polyclonal monospecific antibodies (immunopurified by affinity with the peptide 246–264) and then incubated with the secondary antibody Alexa Fluor 488 goat anti-mouse IgG or the secondary antibody Alexa Fluor 568 goat anti-rabbit IgG, respectively. Control refers to the reaction with the secondary antibodies only. B) Phosphatase activity in cytosolic extracts was determined in a population of 1×10^7^ epimastigotes in assay buffer with or without EGTA, Mn^2+^ and Ca^2+^ in accordance with the manufacturer's instructions (Calcineurin Cellular Activity Assay Kit, Colorimetric, Calbiochem). Data are represented as means ± SD, the results are representative of three independent experiments.

It has been shown by Moreno *et al.* [14] in clone CL Brener that CaNA is present predominantly in the parasite nucleus. We examined the cellular distribution of TcCaNA2 in epimastigotes of CL strain. To this end, parasites were fixed in paraformaldehyde, permeabilized with 0.5% Triton X-100, incubated with specific antibodies to TcCaNA2 and then processed for immunofluorescence. Figure 6A shows that TcCaNA2 has a predominantly cytoplasmic localization, with a diffuse and/or slightly speckled pattern.

To show that CaN activity was detectable in the parasite cytosol, we performed *in vitro* assays with high-speed cytosolic extracts from metacyclic trypomastigotes and epimastigotes depleted of phosphates and nucleotides. The cytosolic extracts were assayed in the presence or absence of EGTA to determine the contribution of calcium-dependent activity (CaN/PP2B) to total phosphatase activity (PP1+PP2A+PP2B+PP2C). In both parasite forms CaN activity was detected in the cytosol, amounting to 34.6% and 40.0% of the total phosphatase activity in metacyclic forms and epimastigotes, respectively. Figure 6B shows the result on CaN activity in cytosol of epimastigotes.

### Inhibition of *T. cruzi* proliferation and host cell invasion by antisense TcCaNA2 oligonucleotides

**Figure 7:**
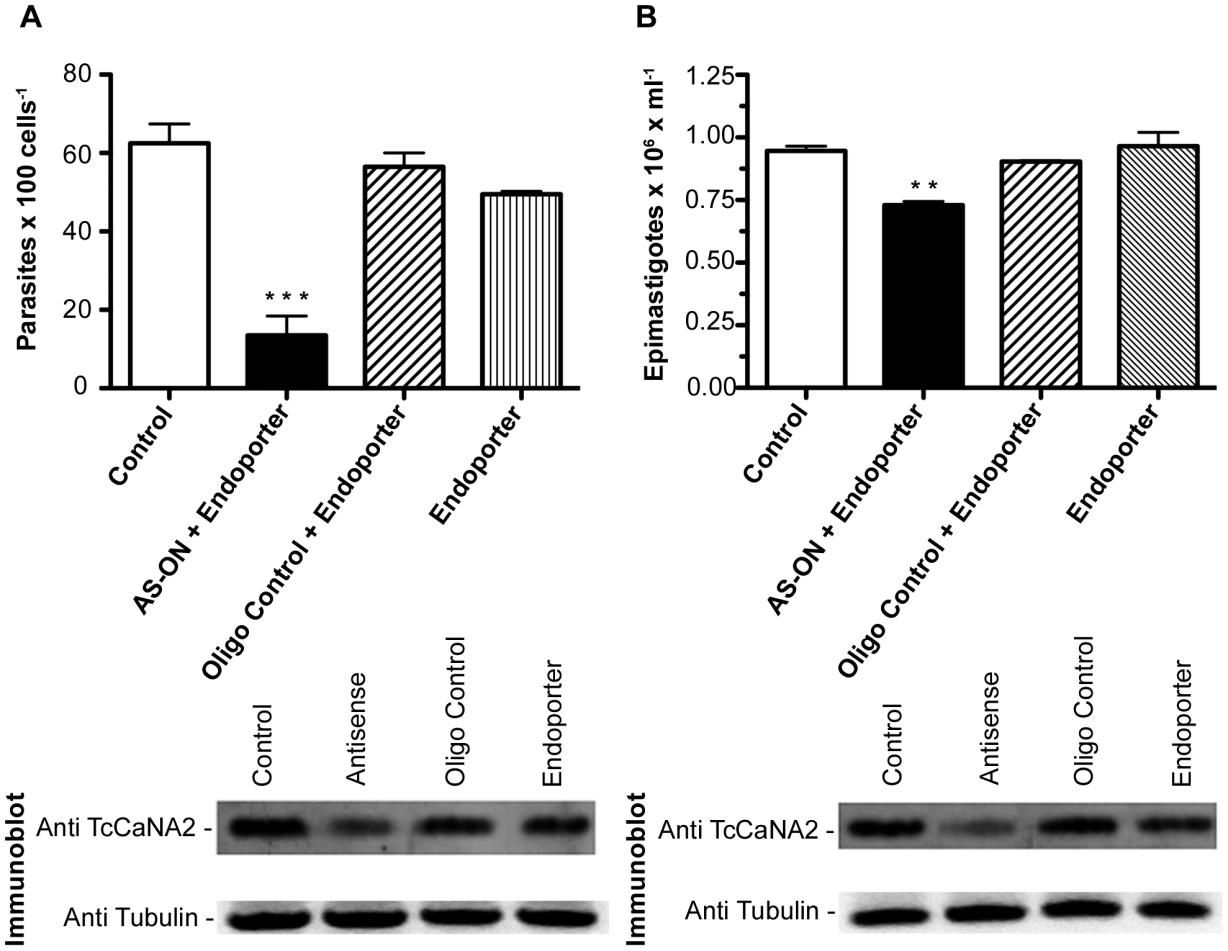
Inhibitory effect of antisense TcCaNA2 oligonucleotides on *T. cruzi* cell invasion and proliferation. A) Upper panel: MT treated for 24 h with the AS-ONs directed to TcCaNA2 and then incubated with HeLa cells for 3 h at 37**°**C. After washes with PBS and staining with May-Grünwald Giemsa, the number of intracellular parasites in a total of 100 cells was counted. Values are the means ± SD of at least three independent experiments performed in triplicate. Statistical significance was determined by the Tukey test (*p*<0.05) ***p = 0.0008. The lower panel shows the analysis of TcCaNA2 expression in the whole cell lysates detected by immunoblotting with a polyclonal antibody against TcCaNA2. In the lower panel, equal protein loading was checked by immunoblotting with mouse anti-alpha tubulin. B) Upper panel: epimastigotes were cultured in 96-well microplates in the presence or absence of AS-ONs. Cell counts were performed after 72 hours using a Neubauer hemocytometer. Values are the means ± SD of at least three independent experiments performed in triplicate. Statistical significance was determined by the Tukey test (*p*<0.05) **p = 0.0038. The lower panel shows the analysis of TcCaNA2 expression in the whole cell lysates detected by immunoblotting with a polyclonal antibody against TcCaNA2. In the lower panel equal protein loading was checked by immunoblotting with mouse anti-α tubulin.

We had previously found that CaN is implicated in host cell invasion [15]. To determine if TcCaNA2 was involved in that process, we performed an inhibition assay using third-generation AS-ONs (morpholino oligonucleotides) associated with the Endo-Porter carrier (Gene Tools). This strategy has been used successfully in many studies [15], [25]–[28]. Metacyclic trypomastigotes (4×10^7^) were incubated with 10 µM of sense or antisense morpholino oligonucleotides directed to TcCaNA2 for 24 h and then incubated with HeLa cells for 3 h to analyze their invasive capacity. After washing with PBS, cells were fixed and stained with Giemsa, and the number of intracellular parasites was counted. Parasites treated with antisense TcCaNA2 oligonucleotides showed a significant decrease in invasivity (70%) while no inhibitory effect of sense oligonucleotides on parasite infectivity was observed (Figure 7A). The decrease in TcCaNA2 expression in parasites treated with antisense oligonucleotide but not with their sense counterparts was ascertained by immunoblotting using anti-TcCaNA2 antibodies, 45% according to densitometric analysis (Figure 7A, lower panel).

To demonstrate that the reduced cell invasion capacity of metacyclic forms treated with antisense oligonucleotides was not due to diminished parasite viability, we performed an additional assay that consisted of determining the parasite's capacity to migrate through a polycarbonate transwell filter coated with gastric mucin. Viable parasites cross the gastric mucin-coated filter propelled by ATP-driven flagellar movement [20]. Metacyclic forms were treated as above with 10 µM of sense or antisense morpholino oligonucleotides directed to TcCaNA2 with Endo-Porter or with the carrier alone. After 16 h at 28°C, the parasites were used in a parasite migration assay through a mucin layer and in a cell invasion assay. Gastric mucin-coated transwell filters were placed onto the wells of 24-well plates containing metacyclic forms in PBS (10^7^/mL), and 100 µL PBS were added to the filter chamber. After 1 h at 37°C, 10 µL were collected from the filter chamber to determine the number of parasites. We found that a comparable number of parasites had crossed the gastric mucin-coated filter regardless of whether they had been pretreated with sense or antisense oligonucleotides and Endo-Porter or with Endo-Porter alone (Figure S1), similar results were observed by Trypan blue exclusión. Although all parasites exhibited high motility, only those treated with antisense oligonucleotide and Endo-Porter had their capacity to invade HeLa cells diminished (by about 75%). Untreated parasites or those treated with sense oligonucleotides and Endo-Porter or with Endo-Porter alone did not show a reduction in their ability to invade these cells.

As epimastigote proliferation was reduced by CaN inhibitors (Figure 1), we examined the contribution of TcCaNA2 in the process using the antisense strategy as above. Epimastigotes in exponential growth phase were treated or not with sense or antisense oligonucleotides against TcCaNA2 for 72 h. Parasites treated with antisense TcCaNA2 oligonucleotides showed a slight decrease in their proliferative capacity (Figure 7B). The decrease in TcCaNA2 expression in epimastigotes treated with antisense oligonucleotides but not with their sense counterparts was ascertained by immunoblotting using anti-TcCaNA2 antibodies, 55% according to densitometric analysis. As shown in Figure 7B (lower panel), there was a marked decrease in TcCaNA2 expression when epimastigotes were treated with antisense oligonucleotides. If TcCaNA2 played a critical role in epimastigote replication, such a decrease would have had a greater effect on proliferation.

## Discussion

*T. cruzi* calcineurin (TcCaN) has been shown to play a role in host cell invasion [15]. Recently, Kulkarni *et al.* [29] demonstrated that parasites exposed to cyclophilin-trialysin exhibit enhanced binding to and invasion of host cells, leading to higher infectivity via calcineurin activation. In this study we found that TcCaN is also implicated in the process of epimastigote replication. Furthermore, existing knowledge about Ca^2+^-dependent phosphatase TcCaN was further enhanced by characterization of the gene coding for a new isoform of TcCaNA, the catalytic subunit A, which exerts its activity through its association with the Ca^2+^-binding regulatory subunit B (CaNB).

Unlike the CaNA homologous protein described by Moreno *et al.* [14], which is predominantly localized in the parasite nucleus, TcCaNA2 is cytoplasmic. TcCaNA2 contains two functional domains: a catalytic domain homologous to the protein phosphatase 2A and the domain that interacts with TcCaNB, both domains characteristic of previously reported CaNA homologs [14], [15]. The lack of a calmodulin-binding domain in TcCaNA2 indicates that its activity is independent of calmodulin. Compatible with this was the finding that TcCaNA2 activity is enhanced by Mn^2+^ rather than by Ca^2+^. The catalytic domain of PPP (phosphoprotein phosphatase) has the phosphoesterase consensus motif, with three conserved motifs in separate regions showing the configuration **D**X**H**(X)_n_ G**D**XXDR(X)_m_ G**N** HD/E [30], [31]. Although there are mutations in the catalytic site of TcCaNA2, suggesting that it may behave as a pseudophosphatase [32], arguments against this idea include the existence in TcCaNA2 of histidine (H) in the G**N**HE domain, which acts as a proton donor in the catalysis [33], in addition to the four amino acids involved in metal coordination, of which only one is non-conserved. The recombinant protein TcCaNA2 showed enzymatic activity using *p*-NPP as substrate. Also, near the C-terminal portion of the TcCaNA2 sequence there is the highly conserved SAPNY motif, which is “conventional” in eukaryotic PPP, tyrosine (Y) being implicated in the interaction with regulators and inhibitors [34]–[37]. This is in contrast to what is found in other catalytic subunits of CaN [for instance the α isoform of rat CaNA, in which a leucine (L) residue is present in the SAPNYL motif making it more susceptible to okadaic acid, a characteristic of phosphatases PP1 and PP2A] [38]. Together with the configuration of the invariant PPP motifs in TcCnA-like, which adjusts to the configuration -GDXHG-, -GDXVXRG-, -GNH- [14], the differences between TcCnA-like and TcCaNA2 in the hydrophobic profiles of the domain that interacts with TcCaNB suggest that these two calcineurin-type protein phosphatases may play distinct functional roles (data not shown). This is supported by their different subcellular localization. Multiple sequence alignment of TcCaNA2 with catalytic subunits of calcineurin from other organisms, revealed a 20 residues long stretch that is found only in trypanosomatids, whose composition in *T. cruzi* is 243-VSGGSGSDYYTPSAGPSYGS-262 (Figure 2A). The functional relevance of this sequence is not known. Bioinformatics tools associated the sequence with two motifs: one the NURR type present in orphan nuclear receptors, and the other associated with calpain-type cysteine proteases (data not shown).

Between one third and one half of all enzymes described to date must associate with metals to perform their function [39]. In the case of calcineurin, which belongs to the hydrolase class (EC Number 3.1.3.16) and whose systematic name is phosphoprotein phosphohydrolase, Fe^3+^ and Zn^2+^ have been described as cofactors in its active site [40]–[42]. Our results showed that, under the conditions assayed, the recombinant TcCaNA2 is activated by Mn^2+^ and Ni^2+^, with no substantial activation by Mg^2+^ or Ca^2+^. By sequentially removing the non-catalytic domains of CaN, such as the calmodulin binding domain and autoinhibitory domain, Liu *et al.* [24] observed increases in phosphatase activity, clearly demonstrating that non-catalytic domains negatively regulate the activity of the enzyme and act as intra-molecular inhibitors. This sequential domain deletion favors CaN activation by Mn^2+^/Ni^2+^ but not by Mg^2+^, suggesting that enzyme activation by Mn^2+^/Ni^2+^ is mainly mediated via the catalytic domain [24]. Our finding that TcCaNA2 lacks the calmodulin-binding domain and the autoinhibitory domain is consistent with its activation by Mn^2+^/Ni^2+^. Assays using the recombinant TcCaNA2 showed that the enzyme can function when dissociated from the regulatory subunit TcCaNB. However, it should be borne in mind that inside the parasite TcCaNA2 is bound to the Ca^2+^-binding regulatory subunit TcCaNB (Figure 5) and that the enzyme is activated during *T. cruzi* invasion of host cells, a process associated with an increase in cytosolic Ca^2+^ concentration. Because of the presence of a highly hydrophobic CaNB domain, which is located between the catalytic and the calmodulin-binding domains, CaNA and CaNB subunits form a heterodimer [43]. This interaction can only be dissociated by strong denaturing agents such as urea at 6 M [44]. We found that 6 M urea dissociates TcCaNA2 from TcCaNB.

Calcineurin contributes in a variety of cellular signaling events and activation processes [45]. In eukaryotic pathogens, it has been associated in the regulation of specific steps of the cell cycle; in intracellular *Toxoplasma gondii*, the loss of host cell potassium, activates a phospholipase C that, in turn, causes an increase in cytoplasmic [Ca^2+^] causing the parasite output from host cell, by the activation of at least two signaling pathways: the protein kinase and calcineurin [46]. Similarly, in *T cruzi*, entry into the host cell is a process dependent [Ca^2+^], recent studies show that calcineurin, specifically, the regulatory subunit CaNB is present in this parasite (TcCaNB) and is involved in the process of invasion of target cells. Treatment of parasites with antisense phosphorothioate oligonucleotides directed to TcCaNB, which reduced the expression of TcCaNB and affected TcCaN activity, resulted in ∼50% inhibition of HeLa cell entry by MT or TCT [15].

Kumar *et al.*, [47], show that CsA inhibits the intraerythrocytic replication of *P. falciparum* and that both, Cyp19A and Cyp19B, are potent effectors of CsA-mediated inhibition of recombinant *P. falciparum* CaNA *in vitro*. CsA-resistant parasites, isolated from erythrocytic cultures, contained mutations in the CaNA and CaNB subunits and in Cyp19A and Cyp19B. Using Geldanamycin (GA), an inhibitor of plasmodial Hsp90 [48], [49], they also show that parasitic Hsp90 is associated with CaN, strongly suggesting that Hsp90 regulates CaNA folding and hence regulates all cellular events that require the phosphatase activity of calcineurin, so the antimalarial activities of CsA and GA would be synergistic. Potenza *et al.*, [50], studied cDNA clones encoding cyclophilin isoforms in epimastigotes of *T. cruzi*. These genes were also detected in amastigotes and trypomastigotes. Four cyclosporin A-binding proteins were isolated in epimastigote extracts, which were identified by mass spectrometry as TcCyP19, TcCyP22, TcCyP28 and TcCyP40, these cyclophilins of *T. cruzi* would be of importance to the mechanism of action of CsA. In the present study, CsA and others calcineurin inhibitors, inhibit the invasion and proliferation processes.

On the other hand, calcineurin is involved in the morphogenesis and virulence of multiple pathogenic fungi: in *Candida* spp., calcineurin participates in antifungal drug resistance/tolerance, survival in serum, and virulence [51]–[54]; in *Paracoccidioides brasiliensis*, calcineurin plays a role in morphogenesis [55]; and in *Aspergillus fumigatus*, calcineurin regulates morphogenesis and thereby pathogenesis [56]. In addition, calcineurin is essential for growth at elevated temperatures in the human fungal pathogen *Cryptococcus neoformans* [57]. Studies conducted in the protozoan parasite *Leishmania major* suggest that Ca^2+^ influx and activation of calcineurin signaling is required for parasite differentiation and adaptation to cellular stress encountered (elevated temperatures) during infection of the mammalian host [58]. In this study, the disruption of calcineurin function, achieved by deletion of the gene encoding the CnB subunit, had no effect on promastigote growth at 27°C or the development of infectious metacyclic promastigotes in stationary-phase cultures. However, disruption of calcineurin function was associated with a marked increase in the sensitivity of promastigotes to elevated temperature and perturbations in membrane lipid composition. In our study, the temperature changes do not affect the infective form (MT) in motility or viability, both ATP-dependent processes [20], as demonstrated by the parasite migration assay through gastric mucin layer. The decrease of the invasive capacity of metacyclic forms, treated and not treated with antisense oligonucleotides at 28°C, was not due to diminished viability, because the parasites that were incubated at 37°C crossed through a polycarbonate transwell filter coated with gastric mucin, demonstrating that are not affected by the temperature stress. TcCaNA2 is expressed in all *T. cruzi* developmental forms (Figure 3D). In metacyclic trypomastigotes, the decrease in TcCaNA2 expression brought about by the use of a TcCaNA2-targeted anti-sense strategy resulted in reduced capacity to invade host cells. In previous studies, the anti-sense approach directed to inhibit TcCaNB, the regulatory subunit with EF-Hands Ca^2+^-linking motifs, rendered metacyclic forms as well as tissue culture-derived trypomastigotes less infective toward target cells [15]. It was also demonstrated that *T. cruzi* protein dephosphorylation by TcCaN is in fact associated with a decrease in parasite internalization assuming that treatment of metacyclic forms with TcCaN inhibitor CsA, which diminishes the phosphorylation levels of serine/threonine residues of high-molecular-weight proteins, inhibits host cell invasion [15]. An expansion of the serine/threonine phosphatase family and a low proportion of tyrosine phosphatases have been found in *T. cruzi* compared with other eukaryotic genomes [32].

Another role of TcCaN is its involvement in parasite multiplication. TcCaN inhibitor CsA affected epimastigote multiplication, confirming the data reported by Búa *et al.* [59]. CsA probably binds to the cytosolic protein cyclophilin (CyP), forming a complex that, through its association with the invariant regulatory subunit TcCaNB, would inhibit TcCaN in a manner similar to that of its mammalian counterpart [60]. Members of the CyP family identified in *T. cruzi*, called peptidyl-prolyl *cis/trans* isomerases, have their activity inhibited by CsA and its analogs [59], and the affinity of CyP for CsA has been documented [61]. It appears that the involvement of TcCaNA2 in epimastigote proliferation is partial, as judged by the weak inhibitory effect on epimastigote replication following treatment with antisense oligonucleotides against TcCaNA2. It is possible therefore that the major contribution to epimastigote replication comes from TcCaNA bound to TcCaNB rather than from TcCaNA2/CaNB.

Assuming that *T. cruzi* expresses TcCaNA and TcCaNA2, both of which can associate with TcCaNB, one interesting possibility is that the two isoforms are engaged in distinct events during the parasite life cycle. TcCaNA2/TcCaNB present in *T. cruzi* cytosol would be predominantly activated in the infective trypomastigote forms, leading to dephosphorylation of serine/threonine residues of proteins implicated in cell invasion. In favor of this view is the fact that a partial decrease in TcCaNA2 expression resulted in a significant reduction in metacyclic trypomastigote internalization, a short process involving signaling events in the cytosol, whereas TcCaNA/TcCaNB may be activated in replicative epimastigote forms to promote parasite proliferation, which implicates transcription in the nucleus [62]. In addition to being inhibited by CsA, epimastigote multiplication is inhibited by FK506 and INCA-6, which are also CaN inhibitors. In a mechanism similar to that associated with CsA, FK506 exerts its inhibitory effect by forming a drug-immunophilin complex with CaN [63], whereas the mechanism of action of INCA-6 is linked to blocking of the substrate recognition site by a covalent union to CaN, inhibiting dephosphorylation of nuclear factor of activated T cells (NFAT) and interrupting the formation of the CaN-NFAT complex [64]. Similarly, kaempferol, binds directly to the catalytic site in CaNA interacting with Leu 312 [65]. Our results showed no inhibition in proliferation and cell invasion in parasites treated with kaempferol. These findings are consistent with the absence of Leu 312 in TcCaNA2 (Figures 1B and 2A).

The mechanism involving phosphorylation/dephosphorylation events that play an important role in cell cycle progression, may operate in *T. cruzi* epimastigotes. It is of note that *T. cruzi* TcCnA-like protein has been detected predominantly in the nucleus of this parasite [14]. TcCaNA2/CaNB and TcCaNA/CaNB, which have distinct cellular localizations, may play a critical role at different stages of *T. cruzi* development.

Many authors suggest calcineurin as a potential chemotherapeutic target against pathogenic; fungi, helminths and protozoa [46], [47], [66]–[70]. Our revision strongly supports that TcCaNA2 is a good candidate for chemotherapeutic target given the differences with its human counterpart. In fact, the *T. cruzi* calcineurin does not possess the calmodulin binding domain and the autoinhibitory domain which are present in the human enzyme. Besides this, TcCaNA2 presents a 20 amino acids long stretch (243-VSGGSGSDYYTPSAGPSYGS-262) in the catalytic domain that is absent in the human calcineurin and is conserved in all trypanosomatids with minimal differences. Also, comparative sequence analysis shows only 44% of identity between human calcineurin and TcCaNA. Taking into account that TcCaNA2 differs considerably in its primary structure from human CaNA and that it may play a key role in host cell invasion by *T. cruzi*, it should be considered a potential target for chemotherapeutic intervention in Chagas disease.

## Supporting Information

Figure S1 **Effect of morpholino antisense oligonucleotides on migration of** ***T. cruzi*** **metacyclic forms through gastric mucin.** Parasites, previously maintained for 16 h in PBS at 28°C with morpholino sense or antisense oligos plus endoporter or with endoporter alone, and the non treated controls were added to the bottom of 24-well plates. Then polycarbonate transwell filters coated with gastric mucin were placed onto parasite-containing wells. After 1 h incubation at 37°C, samples from the filter chamber were collected and the numbers of parasites counted. Results were expressed as mean ± standard deviations of the three independent experiments performed in triplicate.
(TIFF)
