# Expression of Concern: A Cytoplasmic New Catalytic Subunit of Calcineurin in *Trypanosoma cruzi* and Its Molecular and Functional Characterization

**DOI:** 10.1371/journal.pntd.0013162

**Published:** 2025-06-04

**Authors:** The PLOS Neglected Tropical Diseases Editors

After this article [1] was published, concerns were raised about Figures 3, 5 and 7.

Specifically:

- When levels are adjusted to visualize the background, there appear to be vertical discontinuities in the following panels:
  - In panel 1 of Figure 3B, between lanes 1-2.
  - In panel 1 of Figure 3C, between lanes 1-2.
  - In the Immunoblot Anti TcCaNA2 panel of Figure 3D, between lanes 2-3.
  - In the Anti TcCaNA2 panel of Figure 7A, between lanes 3-4.
  - In the Anti TcCaNA2 panel of Figure 7B, between lanes 1-2, lanes 2-3, and lanes 3-4.
- In Figure 5B, when levels are adjusted to visualize the background, there are regions in the background that appear similar between lanes 1 and 2.

The first author provided the original image underlying Figure 5A and 5B, available in S1 File. PLOS assessed Figure 5B and considers the background regions to be similar rather than identical. In addition, as no bands are present in the regions of background similarity in S1 File, PLOS considers this concern to be resolved.

Regarding the concerns raised for Figure 3B, the first author stated that an error occurred during figure preparation, leading to the superimposition of lanes 1 and 2. They provided both original and repeat data from the time of the original experiments for Figure 3B(2), available in S2, S3 Files. For Figure 3C, only repeat data for Figure 3C(2) was provided (S4 File), stated to be performed in parallel with the original experiments. The first author also provided repeat data for lanes 2 and 4 of the Anti TcCaNA2 panel in Figure 3D (S5 File), stated to be from experiments carried out at a later date. PLOS considers that the available repeat data provides support for the published results, but does not fully resolve the concerns with Figures 3 and 7.

The first author stated that no original image data are available for Figure 3B(1), Figure 3C, Figure 3D and Figure 7, but they provided the quantitative data underlying the bar graphs in Figure 7 (S6 File). The first author confirmed that the remainder of the underlying data are also unavailable.

In light of the above concerns, which cannot be fully resolved in the absence of all original underlying image data for the panels of concern, the *PLOS Neglected Tropical Diseases* Editors issue this Expression of Concern.

## Supporting information

S1 File Far-western blotting original data underlying Figures 5A and 5B. Original scanned PVDF-membrane.
(JPG)

S2 File Chromoblot TcCaNA2 CLB, CL & G Strains - original data underlying Figure 3B(2). Corresponds to the result obtained from the hybridization of the TcCaNA2 probe on different strains of *T. cruzi*. The G strain data was not presented in the published figure.
(JPG)

S3 File Chromoblot TcCaNA2 CL & G Strains - repeat data supporting Figure 3B(2). Corresponds to the result obtained from the hybridization of the TcCaNA2 probe on different *T. cruzi* strains. The G strain data was not presented in the published figure.
(JPG)

S4 File Northern blot TcCaNA2 with RNA ladder - repeat data supporting Figure 3C(2). Corresponds to one of the results obtained from the hybridization of the TcCaNA2 probe on different cell forms of *T. cruzi.*
(JPG)

S5 File Immunoblot for Anti TcCaNA2 EPI & MT – repeat data supporting Figure 3D. Corresponds to the Western blot analysis of TcCaNA2-CL expression on epimastigotes (EPI) and metacyclic (MT). Immunodetection of total proteins (10 μg) in extracts of EPI and MT of *T. cruzi* was made using polyclonal antibodies directed towards TcCaNA2-CL. The immunoreaction was revealed using a peroxidase-labeled mouse IgG anti-Fc secondary antibody and revealed by DAB (diaminobenzidine).
(JPG)

S6 File Quantitative data underlying bar graphs in Figure 7. Inhibitory effect of antisense TcCaNA2 oligonucleotides on *T. cruzi* cell invasion and proliferation.
(XLSX)
