# Supplementary figures and images for: A Cytoplasmic New Catalytic Subunit of Calcineurin in Trypanosoma cruzi and Its Molecular and Functional Characterization
Source: PLoS Negl Trop Dis. 2014 Jan 30;8(1):e2676. doi: 10.1371/journal.pntd.0002676 (PMC3907409; doi:10.1371/journal.pntd.0002676)

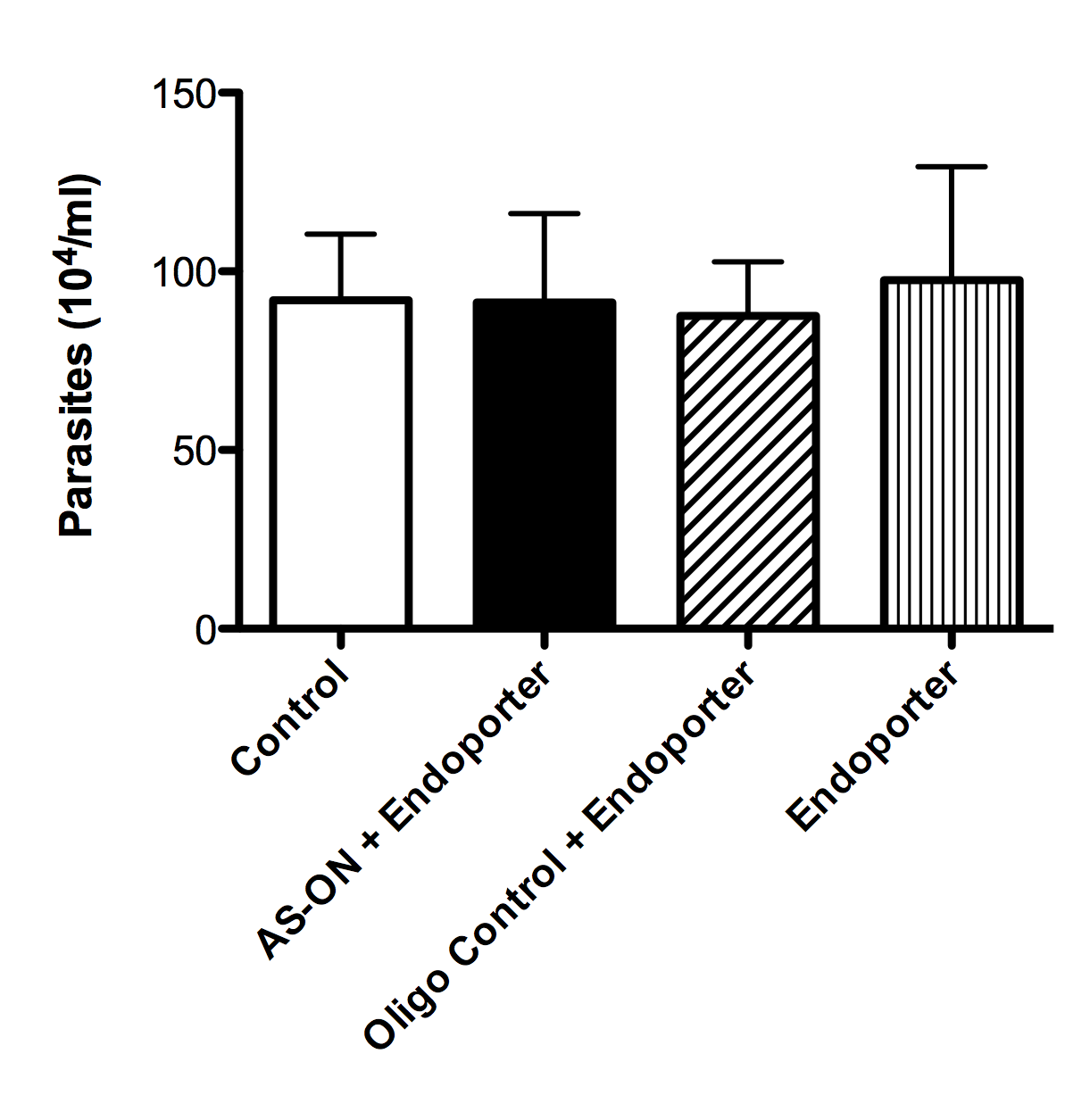

Supplement: Figure S1 — Effect of morpholino antisense oligonucleotides on migration of T. cruzi metacyclic forms through gastric mucin. Parasites, previously maintained for 16 h in PBS at 28°C with morpholino sense or antisense oligos plus endoporter or with endoporter alone, and the non treated controls were added to the bottom of 24-well plates. Then polycarbonate transwell filters coated with gastric mucin were placed onto parasite-containing wells. After 1 h incubation at 37°C, samples from the filter chamber were collected and the numbers of parasites counted. Results were expressed as mean ± standard deviations of the three independent experiments performed in triplicate. (TIFF) [file pntd.0002676.s001.tiff]
