# Supplementary figures and images for: Expression of Concern: A Cytoplasmic New Catalytic Subunit of Calcineurin in Trypanosoma cruzi and Its Molecular and Functional Characterization
Source: PLoS Negl Trop Dis. 2025 Jun 4;19(6):e0013162. doi: 10.1371/journal.pntd.0013162 (PMC12136285; doi:10.1371/journal.pntd.0013162)

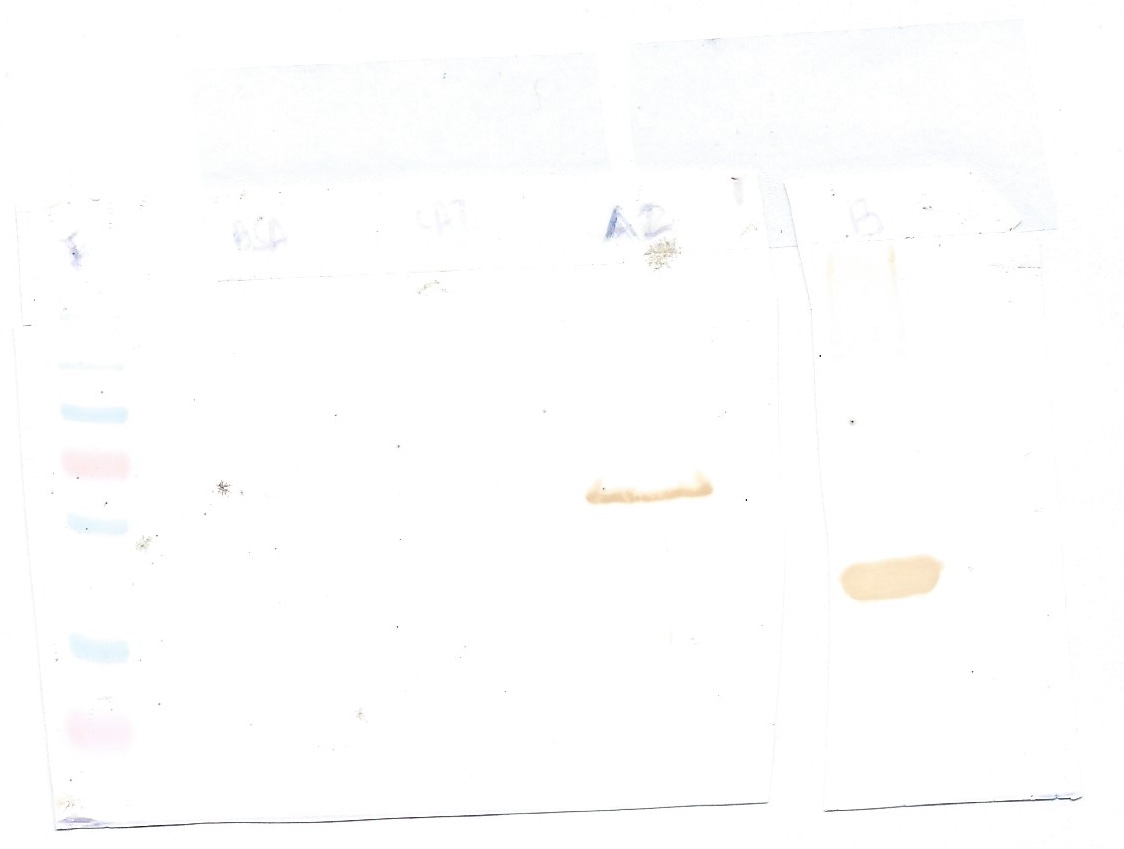

Supplement: S1 File — Original scanned PVDF-membrane. (JPG) [file pntd.0013162.s001.jpg]

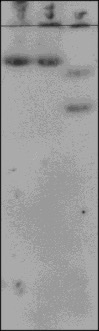

Supplement: S2 File — Corresponds to the result obtained from the hybridization of the TcCaNA2 probe on different strains of T. cruzi. The G strain data was not presented in the published figure. (JPG) [file pntd.0013162.s002.jpg]

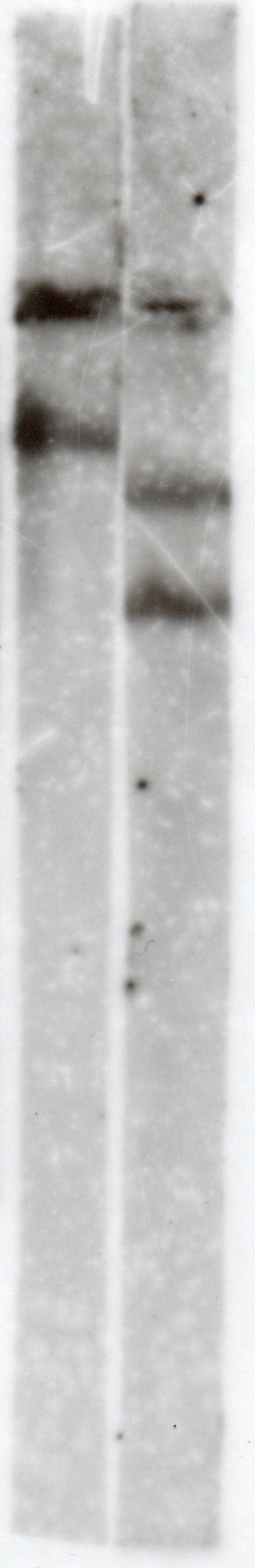

Supplement: S3 File — Corresponds to the result obtained from the hybridization of the TcCaNA2 probe on different T. cruzi strains. The G strain data was not presented in the published figure. (JPG) [file pntd.0013162.s003.jpg]

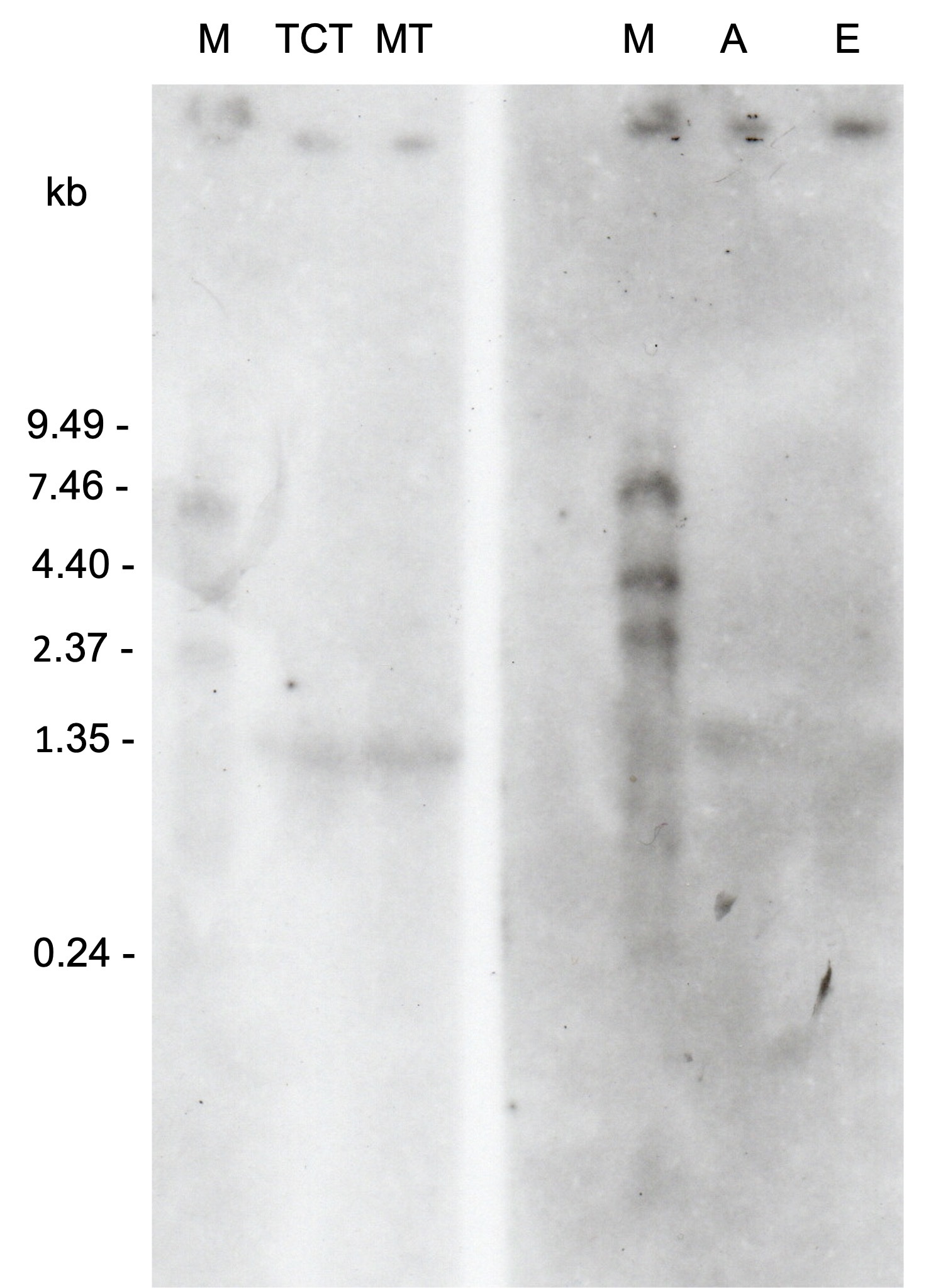

Supplement: S4 File — Corresponds to one of the results obtained from the hybridization of the TcCaNA2 probe on different cell forms of T. cruzi. (JPG) [file pntd.0013162.s004.jpg]

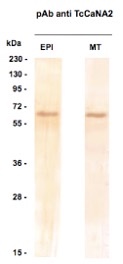

Supplement: S5 File — Corresponds to the Western blot analysis of TcCaNA2-CL expression on epimastigotes (EPI) and metacyclic (MT). Immunodetection of total proteins (10 μg) in extracts of EPI and MT of T. cruzi was made using polyclonal antibodies directed towards TcCaNA2-CL. The immunoreaction was revealed using a peroxidase-labeled mouse IgG anti-Fc secondary antibody and revealed by DAB (diaminobenzidine). (JPG) [file pntd.0013162.s005.jpg]
